# Editorial: Postharvest Ripening, Senescence, and Technology

**DOI:** 10.3389/fgene.2022.920584

**Published:** 2022-06-06

**Authors:** Tie Liu, Karin Albornoz, Angelos Deltsidis, Diane M. Beckles

**Affiliations:** ^1^ Horticultural Sciences Department, University of Florida, Gainesville, FL, United States; ^2^ Departamento de Producción Vegetal, Facultad de Agronomía, Universidad de Concepción, Concepción, Chile; ^3^ Department of Horticulture, University of Georgia, Athens, GA, United States; ^4^ Department of Plant Sciences, MS3, University of California, Davis, Davis, CA, United States

**Keywords:** postharvest, ripening, signalling, senescence, quality

## Introduction

A primary goal of postharvest research is to alter the naturally occurring ripening and senescence processes in harvested products, to amplify those that dictate desirable attributes, while minimizing those that do not. Traits that define quality and indicate the shelf-life of produce are often judged through a multisensorial assessment of the harvested tissue’s physiological state (Shipman et al., 2021). In this Research Topic, the authors used modern genomic tools to understand or manipulate the biological pathways underlying postharvest phenotypes, to enhance outcomes for the consumer. For example, yellowing, a visual proxy for senescence was studied in broccoli by Aghdam et al. and in the model species tobacco by Qin et al. Aroma volatiles, primary indicators of flavor in many fruits and vegetables were investigated in grapes by Zheng et al. Firmness, a key determinant of shipping and handling requirements, shelf-life, and consumer acceptability was the focus of apple improvement described by Migicovsky et al. and also, in the development of a harvest index for persimmon (Yadav et al.). Finally, in strawberry, a high value crop that requires careful handling, the genetic basis of susceptibility to a fungal disease was studied (Chandra et al.). The knowledge from these studies can be applied to improve breeding, postharvest chemical treatments, or the accurate prediction of quality using biomarkers.

## Visual Quality

Yellowing of broccoli due to chlorophyll breakdown is a common manifestation of produce senescence, causing consumer rejection and loss of commercial value, which in turn contributes to postharvest waste and loss (Yang et al., 2022). Aghdam et al. reported that the exogenous application of phytosulfokine-α (PSKα), a signalling peptide and growth factor, delayed senescence in broccoli florets during low temperature postharvest storage. PSKα treatment at 150 nM reduced yellowing by retarding chlorophyll degradation, decreased ethylene production, and promoted higher endogenous accumulation of hydrogen sulphide (H_2_S), a signalling molecule associated with delayed senescence. This work not only provides a potential practical solution to address quality, but opens the possibility of understanding the signal transduction pathways underlying yellowing of broccoli florets.

Tobacco is a functional genomics model for the study of leaf processes including senescence. Qin et al., focused on the tobacco Golden2-like (GLK) transcription factor family, that has roles in chloroplast formation, development, and aging. They identified six GLK genes that were expressed synchronously with leaf senescence, implicating these GLKs in control of elements of dark-induced senescence. These data are relevant to the study of postharvest yellowing of leafy greens and members of the brassicas, as their phylogenetic analysis suggests functional conservation of GLKs.

## Aroma

“Chardonnay” is a popular white grape (*Vitis vinifera*) variety, but it has no typical aroma, which is a major challenge in the production of high-quality white wine. Zheng et al. identified a novel clonal “Chardonnay” variety with a fragrant flesh called “Bud mutation” using inter-simple sequence repeat (ISSR) markers. The mutant fruit matured 10 days earlier than fruit from “Chardonnay,” but more importantly, produced a superior aroma volatile when processed. The mutant had higher levels of linalool, geraniol, acetic acid, butyl ester, and other components of the Muscat fragrance. It also had higher quality components such as carotenoids, sugars, tartaric acid, and malic acids, compared to that of Chardonnay. This clonal variety can be used to deepen our understanding of aroma volatile production in fleshy fruits, and their interconnectedness with other pathways involved in fruit quality.

## Firmness and Shelf-Life

The timing and extent of pre-harvest ripening and postharvest tissue softening are key features for improving fruit quality by breeding. Using Genome Wide Association Studies analysis of apple (*Malus* spp.), Migicovsky et al. identified *NAC18.1*, a NAC transcription factor. *NAC18.1* is an orthologue of the well-characterized *NON-RIPENING (NOR)* gene in tomato, that regulates fruit softening and carotenoid development (Gao et al., 2020). Several *NAC18.1* allelic variants were identified in the coding and promoter regions across 18 apple accessions. Complementation of two *NAC18.1* alleles cloned from apple and expressed in tomato, provided evidence that the function of *NAC18.1* was conserved in these species. These *NAC18.1* polymorphisms may thus underlie substantial variation in apple firmness by modulating a conserved fruit ripening program. This work will enable the selection of genotypes varying in firmness and assist in apple breeding which is challenging as a perennial crop.

Persimmons (*Diospyros kaki*) are consumed either soft or firm, and different markets have preferences for one type or the other. Accurately determining a harvest maturity index for persimmons that delivers the textural quality the market desires would be economically valuable. While the maturity date can provide such a reference point, used alone, it is rarely accurate. Yadav et al. were successful in using a DA-meter, to non-destructively assay fruit chlorophyll levels in 18 varieties of persimmon. Chlorophyll levels were used as a proxy for developmental age, and to provide a reference point for comparing other ripening-associated postharvest quality traits, such as astringency, firmness, and *Alternaria* susceptibility, among others. This work is an important step in determining time-of-harvest as the repercussions for storage-life and consumer acceptance are immense.

Colletotrichum crown rot, caused by the necrotic fungus *Colletotrichum gloeosporioides*, is one of the major diseases of strawberry. It aggressively invades fruit, crown, and leaf tissue, resulting in plant collapse that causes significant economic damage. In this study, comparative transcriptomic analysis was conducted between a crown rot resistant and a sensitive strawberry cultivar to identify disease resistant genes. Three candidate genes near the previously discovered crown rot resistant gene *FaRCg1* are potential markers that can facilitate development of resistant varieties through marker-assisted selection. More resilient plants would promote the maximal resource allocation to fruit production and improve postharvest quality.

## Conclusion

If consumers are presented with high-quality fruit and vegetables that do not spoil quickly, it would increase their satisfaction, reduce waste and loss, and encourage repeat purchases. A multitude of approaches and tools are needed to achieve this goal. The papers in this Research Topic offer a cross-section of the current priorities placed by researchers.

## References

[B1] Gao Y. Wei W. Fan Z. Zhao X. Zhang Y. Jing Y. (2020). Re-evaluation of the Nor Mutation and the Role of the NAC-NOR Transcription Factor in Tomato Fruit Ripening. J. Exp. Bot. 71, 3560–3574. 10.1093/jxb/eraa131 32338291 PMC7307841

[B2] Shipman E. N. Yu J. Zhou J. Albornoz K. Beckles D. M. (2021). Can Gene Editing Reduce Postharvest Waste and Loss of Fruit, Vegetables, and Ornamentals? Hortic. Res. 8, 1. 10.1038/s41438-020-00428-4 33384412 PMC7775472

[B3] Yang Q. Zhou Q. Zhou X. Fang H. Zhao Y. Wei B. (2022). Insights into Profiling of Glucosinolates and Genes Involved in its Metabolic Pathway Accompanying Post-harvest Yellowing of Broccoli. Postharvest Biol. Technol. 185, 111780. 10.1016/j.postharvbio.2021.111780

